# “Effects of the COVID-19 pandemic on university students’ physical health, mental health and learning, a cross-sectional study including 917 students from eight universities in Germany”

**DOI:** 10.1371/journal.pone.0273928

**Published:** 2022-08-31

**Authors:** Sandra Claudia Gewalt, Sarah Berger, Regina Krisam, Markus Breuer

**Affiliations:** 1 SRH University Heidelberg, Heidelberg, Germany; 2 Centre for Postgraduate Nursing Studies, University of Otago, Christchurch, New Zealand; 3 Institute of Medical Biometry, Heidelberg University, Heidelberg, Germany; King Abdulaziz University, SAUDI ARABIA

## Abstract

The COVID-19 pandemic has severely affected physical and mental health. Since its commencement in 2020, social distancing has become the “new normal”. Temporary lockdowns and distance learning have disproportionately affected young adults, including university students. To identify effects of the pandemic on university students’ physical and mental health and learning, this empirical study included eight universities in Heidelberg, Mannheim and Ludwigshafen. Data was collected in May and June 2020. The self-administered survey was filled by 1,246 university students. 917 students completed the survey in full. 80.6% were bachelor students (n = 738), the mean semester was 3.8 and mean age was 23.1 years. 51.8% (n = 472) were female students and 47.4% (n = 432) male students. 38.5% (n = 352) stated a deterioration in physical health and 53.1% (n = 485) in mental health. From 0 to 10, students rated mean levels of stress highest due to social distancing (5.6), spending most time at home (5.0) and e-learning (4.5). Compared to male students, female students’ mental health worsened significantly (58.7% vs. 46.8%). A logistic regression analysis identified gender having a significant effect on university students’ stress levels: males seemed to have a lower risk of moderate to high levels of stress compared to females (odds ratio = 0.698; 95% CI = 0.515 to 0.945). Age, city of university and semester did not show a significant effect. The results are important to both regional and international audiences as university students face similar physical and mental health challenges due to the pandemic and its public health measures. Low-threshold initiatives are needed to mitigate the effects of the pandemic. These may include measures to reinforce students’ locus of control, sense of belonging, relaxation and mindfulness as well as (online) counselling services. Gender-specific differences must be taken into account.

## 1. Background

The COVID-19 pandemic has created a serious threat to short- and long-term physical and mental health as well as social wellbeing. Vulnerable groups, including university students were especially affected. In spring 2020, social distancing became the “new normal” with closed university campuses, remote studies and temporary lockdowns including travel bans. Public life was shut down with non-vital shops, gastronomic businesses and nightclubs being closed and public events were called off. Sports facilities were closed and group sports prohibited. Contact restrictions were implemented. The pandemic caused major health-related risks. Imposed measures to contain its spread restricted individuals’ freedom in an unprecedented way. Health-risks and limitations in individuals’ lives might have affected students’ health and wellbeing adversely.

Due to nation-wide lockdowns, governments closed educational institutions temporarily to help contain the spread of the COVID-19 pandemic. These nationwide closures were impacting almost 70% of the world’s student population [1]. Tertiary education was severely marked by the ban of in-person university lectures and exams, the closing of university facilities including libraries and canteens. These conditions significantly affected young adults including university students, oftentimes causing a more solitary, sedentary, passive and potentially more stressful lifestyle.

### 1.1 Effects on students’ physical health

The viral infection COVID-19 could affect short and long-term physical health. It might cause milder symptoms or in many cases severe illness and death. Typical symptoms included cough, fever, weakness, respiratory difficulties and loss of taste and/or smell [2]. Even though young adults might not be at immediate risk of a fatal outcome, there is increasing evidence on prolonged symptoms, the so called “Long-COVID” [3]. The pandemic poses a risk for students’ short- and long-term physical health.

Yet, students’ physical health was not only directly at risk due to the pandemic but also indirectly due to imposed preventive measures. Social distancing and temporary lockdowns impeded access to physical activities. The prohibition of team sports and the closure of gyms may have caused negative consequences on students’ physical and mental health as there is evidence that physical exercise has a moderate reducing effect on anxiety and can improve mood and mental wellbeing [4]. Staying active with aerobic exercises, body weight training, etc. whilst being at home can help reduce the harmful physical and mental side effects of COVID-19 regulations [5]. A study on female university students in Spain concluded that students who engaged in physical activities had superior physical and emotional self-concept compared to those who did not engage in sports [6]. A recent study on 217 undergraduate students showed that students were more sedentary and reported more symptoms of anxiety and depression compared to previous academic semesters and subsequent academic breaks [7]. Also, a population-based study in Sweden identified that physical inactivity was associated with mental health symptoms [8]. Physical inactivity and stress fuelled a vicious circle potentially causing anxiety and depression [9]. Therefore, insufficient engagement in physical activities is detrimental for both physical and mental health.

### 1.2 Effects on students’ mental health and social wellbeing

Besides the above-mentioned physical health-related aspects, the pandemic potentially affected students’ mental health. The World Health Organization defined mental health as “a state of wellbeing in which the individual realizes his or her own abilities, can cope with the normal stresses of life, can work productively and fruitfully, and is able to make a contribution to his or her community” [10]. Determinants of mental health include social, psychological and biological factors that affect a person’s mental health.

In contrast to mental health, mental or psychological distress is defined as a state of emotional distress or suffering characterized by symptoms of depression [11]. Psychological stress can arise when individuals’ environmental demands strain or exceed their adaptive capacity [12]. Poor mental health can be linked with fast social change, social exclusion, unhealthy lifestyle and physical illness [10].

Resilience has shown itself to be a relevant characteristic to promote and protect mental health and wellbeing. Definitions include the perceived control over one’s life and considering change as positive challenge [13]. Also, the skill to make sense of challenges by focusing on the positive effects or personal development was highlighted [14]. Flourishing takes place when the individual achieves a higher level of performance with the gain of strengthened social relationships [15]. Individuals who are considered resilient deal with challenges in a more positive way, which is protective to mental health. Also, how to activate and foster resilience may play an important role to protect mental health and wellbeing [16]. Research on resilience and the psychological impact on Italian university students during the COVID-19 pandemic found that resilience techniques had beneficial effects on stressful events [17]. Resilience may be an important factor to protect mental health and to empower individuals to overcome difficult times.

Furthermore, ongoing socio-cultural limitations, such as during the pandemic, can pose a risk to students’ mental health and wellbeing. A recent study on university students in Heidelberg, Germany, identified that pandemic-related social restrictions caused a severe reduction in wellbeing in almost three quarter of students [18]. A population-based study found that little social support and significant life events were strongly and independently associated with mental health symptoms [8]. Indeed, social distancing was identified as considerable stressor for students: COVID-19 specific concerns, social isolation, lack of interaction and emotional support as well as physical isolation have been linked to negative mental health trajectories [19]. Social distancing might have also negatively affected the sense of belonging. Belonging seems to have diverse and powerful effects on emotional patterns and cognitive processes. It is often satisfied through friendships and social activities. The lack of attachment is linked to numerous negative effects on health, adaptation and wellbeing [20]. For students’ academic success, the sense of belonging is an important factor [21]. An online survey among Chinese nationals recognized young individuals between 21–40 years as being psychologically more vulnerable during the COVID-19 epidemic compared to older individuals [22]. Also, students were stressed about student dorm clearance and annulation of planned events including exchange studies and graduation ceremonies [23]. A study on in-depth experiences of college students described their feelings as "emotional rollercoaster", accepting a "new normal" and experiencing "growth under pressure" [24]. A rapid review of existing evidence of the COVID-19 on mental health revealed mostly negative psychological effects, including post-traumatic stress symptoms including fear of infection, frustration and boredom [25]. Thus, the pandemic and its accompanying measures including social distancing and nation-wide lockdowns were identified as stressors that could affect students’ mental health negatively.

### 1.3 Gender-related differences

A study on the effects of COVID-19 on students’ mental health in Switzerland showed that female students appeared to have poorer mental health after controlling for various levels of social inclusion and COVID-19-related stressors [19]. These findings were not surprising as research on gender differences related to stress revealed that women perceived more stress than men [26]. A study on stress among medical students associated with the Middle East Respiratory Syndrome-Corona Virus outbreak found that female students had a significantly higher mean stress level than male students [27]. Also, a recent longitudinal study on Chinese adults’ mental health identified that female gender was associated with a higher psychological impact [28].

### 1.4 Effects on students’ learning and grades

Chronic stress, such as during the COVID-19 pandemic, potentially affected students’ health and learning negatively. Increased levels of stress triggered the release of stress hormones, glucocorticoids, that have access to the brain and more particularly to brain regions responsible for memory function, emotions and emotional control [29]. Chronic exposure to elevated levels of glucocorticoids can affect cognition. Stress-related mental illnesses may manifest via burnout, depression or posttraumatic stress disorder [29]. Recent findings on distance learning and students’ health identified that stress significantly lowered learning and psychosocial wellbeing [17]. Thus, chronic stress, as experienced during the ongoing pandemic, might have affected students’ learning and wellbeing negatively.

COVID-19 triggered education institutions to switch to an online-learning system. Shifting from medical school to one’s home triggered feelings of isolation, increased use of email and revealed medical students’ difficulties in creating boundaries between work and home [30]. Also, online exams showed a negative impact on medical sciences students’ habits related to diet, sleep, physical activity and smoking [31]. In contrast, a systematic review and meta-analysis of undergraduate medical education found that there was no proof that offline learning was better compared to online learning [32]. Comparing remote with face-to-face learning in undergraduate students, online and in-person activities can result in similar academic performance [33, 34] and students’ performance, as measured by grade, is independent of the type of learning [35]. Other researchers found that online learning was advantageous in increasing undergraduates’ knowledge and skills [32] and impacted students’ mental state positively [36]. Thus, whilst stress had detrimental effects on learning and wellbeing, online education showed controversial results of potentially positive and negative effects on students’ learning and mental health.

### 1.5 Research question

Based on a literature review, it was identified there was no published investigation in effects of the COVID-19 pandemic on students’ physical and mental health and learning in Germany. Moreover, no research on students’ gender differences and differences between students who perceive high versus low levels of distress was available from the Rhine-Neckar region.

To fill this gap, this study investigated how the COVID-19 pandemic affected university students’ self-reported physical and mental health and academic performance, in Germany. Also, differences related to students’ reporting high versus students reporting low levels of negative stress and differences between male and female students were analysed.

## 2. Methods

This empirical research was based on a cross-sectional study with data being collected during five weeks from end of May until end of June 2020 in the Rhine-Neckar region, in Germany.

### 2.1 Recruitment

Via convenience sampling, the student population in the Rhine-Neckar region was selected. Each of the eight universities’ (of applied sciences) registry offices was approached and asked to send an invitation via email to all students who were currently enrolled. The email included a short description of the purpose of the survey and contained a link to access the survey. A flyer was created and attached to that email.

Additionally, an invitation was posted which included an overview of the survey and a link to access the survey on social media channels of the universities (of applied sciences) and student associations for each university. In a second recruitment wave, a reminder was sent via social media channels.

### 2.2 Participants

Students enrolled at one of the eight universities (of applied sciences) in the three cities in the Rhine-Neckar region, including Heidelberg, Mannheim and Ludwigshafen, were recruited. Due to time and financial constraints, convenience sampling was performed. The overall aim was to include the total student population at the eight universities (of applied sciences) in the Rhine-Neckar region.

Inclusion criteria: Enrolment at one of the eight universities in the Rhine-Neckar region and basic English skills.

The invitation to participate in the survey was solely communicated in English language. Students knew before filling out the survey, that this survey was only offered in English language. Participation was voluntary, the survey took less than five minutes to be completed. Study participation could be ended at any time without any negative consequence. As an incentive, five gift vouchers were raffled with the value of 10 Euro per voucher.

Students were invited to fill the online-based, self-administered survey in English. Data was collected anonymously via an independent service provider. Questions addressed the following areas: Short-term COVID-19-related effects on students’ a) physical and mental health and b) learning. Furthermore, data on the name of university, degree, semester, age, gender, average grade, physical and mental health, fitness, and perceptions of the future was collected. The questionnaire used closed-ended questions with a Likert numerical rating scale.

### 2.3 Data collection

Data was collected during five weeks from end of May 2020 until end of June 2020. Lockdown reached its peak in Germany in March and April 2020. However, daily life was still significantly disrupted by restrictions during May and June. Restrictions included but were not limited to a ban of face-to-face university lectures and the closure of non-essential business sectors.

Data was collected via a questionnaire designed by the researchers based on a literature review. The questionnaire focused on three areas: COVID-19’s perceived effects on students’ physical and mental health as well as learning and grades. The survey was designed striving for a maximum of content validity.

### 2.4 Statistical analysis

The analysis of the survey questions was summarized by number of non-missing values, mean, standard deviation, median, Q1 (25^th^ percentile), Q3 (75^th^ percentile), minimum and maximum (for continuous variables). For binary and categorical variables, absolute and relative frequencies were provided. The number of missing answers was reported as separate category, if available. Percentages were based on all non-missing values (= 100%).

As it is difficult to measure mental health, students’ self-reported levels of negative stress from zero to ten were measured. A value of zero represented “no negative stress” and a value of ten represented “maximum negative stress”. Students’ mental health was assessed by the question “Select your level of negative stress related to the following aspects: mental health (0 = no stress, 5 = medium stress, 10 = highest level of stress)”. To compare students who declared themselves to have no to little stress (from 0 to 4) with students suffering from moderate to high stress (from 5 to 10), a binary mental health variable was derived. To investigate differences between both groups of students, all survey answers were additionally stratified after the binary mental health variable. Both groups were compared using t-tests (for continuous variables), Mann-Whitney u-tests (for ordered categorical variables) or chi-squared tests (for categorical variables).

To investigate the impact of baseline characteristics, such as gender, age, financial situation (university fees and part-time working), degree program, current semester, grades and site on students’ mental health, logistic regression analyses were conducted with the binary mental health variable as outcome variable. For categorical variables, a category is always taken as a reference and results are always set in relation to the reference. The category that does not appear in the table was used as a reference.

Statistical analysis was undertaken using R (version 3.6.3). P-values < 0.05 were regarded as statistically significant. Due to the exploratory nature of the study, derived p-values can only be interpreted descriptively.

### 2.5 Ethics approval

Ethical approval for the research project "Impact of the COVID-19 pandemic on students of the Rhine-Neckar district with a focus on health, finances and learning. A cross-sectional study”was obtained by the Joint Ethics Committee of the Heidelberg University of Education and the SRH University Heidelberg. All open aspects have been addressed and completely answered to the Joint Ethics Committee of the Heidelberg University of Education and the SRH University Heidelberg by SG and MB.

## 3. Results

The self-administered survey was filled by 1,246 students in the Rhine-Neckar region. The following results are based on the analysis of students who filled the survey in full (N = 917).

### 3.1 Baseline characteristics

Baseline characteristics of the sample included parameters that describe general aspects of the students and included city of university (of applied sciences), planned university degree, current semester, age and gender among others.

27.0% (n = 247) of the participants were enrolled in Heidelberg, 25.1% (n = 229) in Ludwigshafen and 47.9% (n = 438) in Mannheim (914 of 917 respondents).

80.6% (n = 738) were enrolled in a bachelor’s degree, 15.9% (n = 146) in a master’s degree, 1.0% (n = 9) pursued a state examination and 2.5% (n = 23) were doctoral students (916 of 917 respondents). In terms of students’ semester, the mean was 3.8 (sd = 2.03) semesters (896 out of 917 respondents). Students’ mean age was 23.1 years (sd = 4.08). Details can be found in Table 1.

**Table 1:** Students’ semester and age.

	What’s your current semester?	Age
N	896	902
missing	21	15
mean	3.8	23.1
SD	2.03	4.08
median	4	22
Q1—Q3	2–6	21–24
min.—max.	1.0–12.0	18.0–54.0

SD: Standard deviation

Respondents’ gender was grouped into female with 51.8% (n = 472), male with 47.4% (n = 432) and divers with 0.9% (n = 8) (912 of 917 respondents).

In terms of students’ degree programs, the Rhine-Neckar region provided a wide variety of study degrees at eight public and private universities (of applied sciences). Five general categories of academic studies were determined: Business, Health, Information Technology, MINT (Mathematics, Computer Science, Science and Technology)/Technical and Other subjects. With 42.8% (n = 392), most of the students were grouped into the Business category. The following three categories each comprised around 16% of the participants: MINT / Technical 16.7% (n = 153), Information Technologies 16.3% (n = 149) and Health 16.2% (n = 148). The “Other subjects” category comprised 8.1% (n = 74) students. This question was answered by 916 out of 917 respondents.

In terms of students’ academic performance, 55.2% (n = 500) stated they generally had good grades, 23.5% (n = 213) said very good grades and 20.4% (n = 185) reported generally having average grades. Eight students (0.9%) disclosed poor grades (906 of 917 respondents).

Every second student in the survey, 52.1% (n = 477) of students, reported being a member of a sports team, fitness club or gym and 5.2% (n = 48) planned to become one. Also, 6.2% (n = 57) would like to be more active but considered costs as being too expensive. Roughly one third of students 36.4% (n = 333) stated they are not a member of a sports team (915 of 917 respondents).

### 3.2 Effects on students’ health

There were considerable effects on students’ physical and mental health which are elaborated in the following sections.

#### 3.2.1 Effects on students’ physical health

38.5% (n = 352) reported a deterioration in their physical health including 4.9% (n = 45) declaring a strong deterioration. 18.0% (n = 165) stated an improvement in their physical health. In terms of overall fitness increases or decreases, 50.4% (n = 461) experienced a decrease in their overall fitness during the pandemic, including 13.0% (n = 119) declaring a strong decrease in overall fitness. In contrast, 25.7% (n = 235) reported an increase in overall fitness. Details can be found in Table 2.

**Table 2:** Change in physical health and fitness.

	Did your physical health improve or worsen during the pandemic?	Did your overall fitness increase or decrease during the current pandemic?
strongly improved	31 (3.4%)	56 (6.1%)
improved	134 (14.6%)	179 (19.6%)
no change	398 (43.5%)	219 (23.9%)
worsened	307 (33.6%)	342 (37.4%)
strongly worsened	45 (4.9%)	119 (13.0%)
missing	2	2

Thus, related to their physical health and fitness, 50% or more students reported a change due to the current pandemic.

#### 3.2.2 Effects on students’ mental health and wellbeing

In terms of students’ mental health, 53.1% (n = 485) reported a worsening of their mental health, among those 9.3% (n = 85) stated a strong worsening. 15.2% (n = 139) stated an improvement of their mental health. A detailed overview is provided in Table 3.

**Table 3:** Change in mental health.

Did your mental health improve or worsen during the current pandemic?	
strongly improved	28 (3.1%)
improved	111 (12.1%)
no change	290 (31.7%)
worsened	400 (43.8%)
strongly worsened	85 (9.3%)
missing	3

To gain insights into students’ mental health, the question “how was your mental health affected by the current pandemic?” was asked and multiple answers could be selected. Almost every second student in this survey reported negative mental health effects (49.8%; n = 445) including feeling sad and/or overwhelmed and/or having poor sleep and/or experiencing lots of negative stress. 4.0% (n = 36) students declared feeling relaxed, balanced, well rested and disposing of lots of positive energy. 13.4% (n = 120) perceived no change of their mental health due to the current pandemic. Details can be found in Table 4.

**Table 4:** Mental health-related feelings.

How was your mental health affected by the current pandemic?	
feeling sad	22 (2.5%)
feeling overwhelmed	29 (3.2%)
poor sleep	26 (2.9%)
poor sleep and feeling sad	20 (2.2%)
lots of negative stress	56 (6.3%)
lots of negative stress and feeling sad	38 (4.3%)
lots of negative stress and feeling overwhelmed	33 (3.7%)
lots of negative stress, feeling overwhelmed and sad	45 (5.0%)
lots of negative stress and poor sleep	30 (3.4%)
lots of negative stress, poor sleep and feeling sad	37 (4.1%)
lots of negative stress, poor sleep, feeling overwhelmed	30 (3.4%)
lots of negative stress, poor sleep, feeling overwhelmed, feeling sad	79 (8.8%)
no change	120 (13.4%)
feeling balanced	22 (2.5%)
feeling relaxed, balanced, well rested and lots of positive energy	36 (4.0%)
other combination	270 (30.2%)
missing	24

As mental health is difficult to measure, the analysis was based on respondents’ perceived stress levels from no stress (0 on rating scale) to maximum stress (10 on rating scale). University students’ level of negative stress was assessed using the question "Select your level of negative stress in relation to the following: mental health (0 = no stress, 5 = medium stress, 10 = highest stress)".

The following levels of distress were based on a comparison of the mean: Negative stress due to social distancing was identified as causing the highest stress factor in students (mean 5.6; sd = 3.00). Thus, a mean of 5.6 displayed high levels of distress. The second highest stress factor in students was due to spending most time at home (mean 5.0; sd = 3.31). Details can be found in Table 5.

**Table 5:** Distress due to social distancing and spending most time at home.

	Negative stress due to social distancing	Negative stress due to spending most time at home
N	910	911
missing	7	6
mean	5.6	5.0
SD	3.00	3.31
median	6	5
Q1—Q3	3–8	2–8
min.—max.	0.0–10.0	0.0–10.0

SD: Standard deviation

The third highest mean was linked to mental health (mean 4.6; sd = 3.09).

These results were followed by negative stress due to closing sports facilities (mean 4.4; sd = 3.47), due to not meeting family mean 4.2 (sd = 3.58) and due to physical health (mean 3.5; sd = 2.88). Lowest levels of negative stress were due to closure of stores that were officially declared non-vital (mean 2.2; sd = 2.56) and due to the closure of student dormitories (mean 1.7; sd = 2.89). Lowest levels of distress were caused the closure of student dorms (mean = 1.7; sd = 2.89) (909 of 917 respondents). Details are displayed in Tables 6 and 7. To summarize, the highest negative stress was due to social distancing, spending most time at home and mental health.

**Table 6:** Distress due to mental health, closing sports facilities and not meeting family.

	Negative stress due to mental health	Negative stress due to closing sports facilities	Negative stress due to not meeting family
N	908	909	905
missing	9	8	12
mean	4.6	4.4	4.2
SD	3.09	3.47	3.58
median	5	5	4
Q1—Q3	2–7	0–7	0–7
min.—max.	0.0–10.0	0.0–10.0	0.0–10.0

SD: Standard deviation

**Table 7:** Distress due to physical health, closing of non-essential shops and student dorms.

	Negative stress due to physical health	Negative stress due to closing shops that are not essential	Negative stress due to closing student dorms
N	911	909	902
missing	6	8	15
mean	3.5	2.2	1.7
SD	2.88	2.56	2.89
median	3	1	0
Q1—Q3	1–6	0–4	0–3
min.—max.	0.0–10.0	0.0–10.0	0.0–10.0

SD: Standard deviation

More than two thirds of study participants, namely 68.6% (n = 626), reported an increase in activities to unwind or to reinforce their mental health (Table 8).

**Table 8:** Change in activities related to relaxation.

Did you increase activities that help you relax or strengthen your mental health?	
very strongly	47 (5.1%)
strongly	114 (12.5%)
a little	356 (39.0%)
very little	109 (11.9%)
not at all	287 (31.4%)
missing	4

As highlighted above, approximately every second student in this survey reported a worsening of their mental health due to the pandemic. Students’ highest mean levels in negative stress were linked to social distancing, spending most time at home and mental health. More than two thirds reported having increased activities to unwind or to reinforce their mental health.

### 3.3 Effects on students’ learning and grades

In terms of the question “How much do you think your learning and education is affected by online classes compared to face-to-face teaching”, students reported mostly negative effects by online classes compared to face-to-face teaching: 23.7% (n = 216) reported a strong decrease in learning and education and 44.2% (n = 403) reported a decrease. Only one in ten students (10.9%; n = 99) reported no change. Almost one in five students (17.7%; n = 161) reported benefits by online classes compared to face-to-face teaching and 3.6% (n = 33) reported even strong benefits (912 of 917 respondents). Details can be found in Table 9.

**Table 9:** Learning via online classes versus face-to-face teaching.

In general, how much do you think your learning and education is affected by online classes compared to face-to-face teaching?	
strongly benefits	33 (3.6%)
benefits	161 (17.7%)
no change	99 (10.9%)
decreases	403 (44.2%)
strongly decreases	216 (23.7%)
missing	5

Related to students’ grades, about one in two students reported no change in their grades during the current pandemic: 53.0% (n = 466). One in three students (33.6%; n = 296) reported a worsening or a strong worsening in their grades. Roughly one in ten students (13.3%; n = 117) reported an improvement in their grades during the pandemic (879 of 917 respondents). Details can be found in Table 10.

**Table 10:** Changes in grades during the pandemic.

In general, how strongly did your grades change during the current pandemic?	
strongly improved	13 (1.5%)
improved	104 (11.8%)
no change	466 (53.0%)
worsened	242 (27.5%)
strongly worsened	54 (6.1%)
missing	38

On a scale from 0 to 10, the level of negative stress due to e-learning via video conferences was reported with a mean value of 4.5 (sd = 3.29) in this study (907 of 917 respondents). The mean value of negative stress due to exams via video conference was rated with 3.8 (sd = 3.69) among students (890 of 917 respondents). Details can be found in Table 14. Thus, students experienced more stress from e-learning than from exams via video conference. Details can be found in Table 11.

**Table 11:** Negative stress due to e-learning and exams via video conference.

	Negative stress due to e-learning via video conference	Negative stress due to exams via video conference
N	907	890
missing	10	27
mean	4.5	3.8
SD	3.29	3.69
median	5	3
Q1—Q3	2–7	0–7
min.—max.	0.0–10.0	0.0–10.0

SD: Standard deviation

### 3.4 Group comparison of students’ stress level

The following sections provide a group comparison of students with no or little negative stress compared to students with moderate to high levels of negative stress. According to the findings, the city of university (of applied science) did not make a significant difference for students with moderate to high negative stress, compared to students with no or low negative mental health-related stress (p = 0.053; chi-squared test). Details can be found in Table 12.

**Table 12:** Comparison of distress and city of university (of applied science).

City of university (of applied science)	Moderate to highest stress level	No or little stress level	P-value
	(n = 491)	(n = 417)	
Heidelberg	123 (25.1%)	123 (29.7%)	0.053
Ludwigshafen	138 (28.1%)	89 (21.5%)	
Mannheim	230 (46.8%)	202 (48.8%)	
missing	0	3	

#### 3.4.1 Health-related criteria and students’ stress level

The following results provide insights into variables such as physical health, fitness, nutrition and mental health and offer a comparison of students reporting moderate to highest levels of distress and students declaring no or little distress.

*3*.*4*.*1*.*1 Physical health-related criteria and students’ stress level*. Findings showed that students with moderate to high negative stress declared more frequently that their physical health worsened (42.8% vs. 22.6%) or even strongly worsened (8.8% vs. 0.5%) since the COVID-19 pandemic, compared to students with no or low negative mental health-related stress (p<0.001; Mann Whitney u-test). Details can be found in Table 13.

**Table 13:** Comparison of distress and changes in physical health.

Did your physical health improve or worsen during the pandemic?	Moderate to highest stress level	No or little stress level	P-value
	(n = 491)	(n = 417)	
strongly improved	7 (1.4%)	24 (5.8%)	<0.001
improved	59 (12.0%)	74 (17.8%)	
no change	172 (35.0%)	222 (53.4%)	
worsened	210 (42.8%)	94 (22.6%)	
strongly worsened	43 (8.8%)	2 (0.5%)	
missing	0	1	

There were significant differences related to the negative stress due to physical health: Students with moderate to highest stress level had a mean of 4.7 (sd = 2.81), compared to the group with no or little stress level (mean 2.0; sd = 2.26; p-value <0.001; t-test). Details can be found in Table 14.

**Table 14:** Comparison of distress due to physical health.

Level of negative stress due to physical health	Moderate to highest stress level	No or little stress level	P-value
	(n = 491)	(n = 417)	
N	491	416	<0.001
missing	0	1	
mean	4.7	2.0	
SD	2.81	2.26	
median	5	2	
Q1—Q3	2–7	0–3	
min.—max.	0.0–10.0	0.0–10.0	

SD: Standard deviation

Students with moderate to highest distress declared more frequently that their fitness strongly worsened (19.8% vs. 5.3%) since the COVID-19 pandemic, compared to students with no or low mental health-related distress (p<0.001; Mann Whitney u-test). Yet, those with no or low mental health-related distress stated their fitness increased (22.8% vs. 16.9%) or strongly increased (8.9% vs. 3.9%) compared to students with moderate to high mental distress. Details can be found in Table 15.

**Table 15:** Comparison of distress and changes in fitness.

Did your overall fitness increase or decrease during the current pandemic?	Moderate to highest stress level	No or little stress level	P-value
	(n = 491)	(n = 417)	
strongly increased	19 (3.9%)	37 (8.9%)	<0.001
increased	83 (16.9%)	95 (22.8%)	
no change	105 (21.4%)	110 (26.4%)	
decreased	187 (38.1%)	152 (36.5%)	
strongly decreased	97 (19.8%)	22 (5.3%)	
missing	0	1	

Students’ distress related to the closure of sports facilities showed that students with moderate to high mental distress had a significantly higher mean of distress (4.9 (sd = 3.55) vs. 3.7 (sd = 3.24)) compared to students with no or low mental health-related distress (p<0.001; t-test). Details can be found in Table 16.

**Table 16:** Comparison of distress and closing of sports facilities.

Level of negative stress due to closing sports facilities	Moderate to highest stress level	No or little stress level	P-value
	(n = 491)	(n = 417)	
N	491	415	<0.001
missing	0	2	
mean	4.9	3.7	
SD	3.55	3.24	
median	5	3	
Q1—Q3	1–8	0–6	
min.—max.	0.0–10.0	0.0–10.0	

SD: Standard deviation

*3*.*4*.*1*.*2 Mental health-related criteria and students’ stress level*. Significant changes in mental health were observed: Students with moderate to high distress declared more frequently that their mental health worsened (64.3% vs. 19.2%) or strongly worsened (16.5% vs. 1.0%) since the COVID-19 pandemic, compared to students with no or low mental health-related distress (p<0.001; Mann Whitney u-test). Detailed information can be found in Table 17.

**Table 17:** Comparison of distress and changes in mental health.

Did your mental health improve or worsen during the current pandemic?	Moderate to highest stress level	No or little stress level	P-value
	(n = 491)	(n = 417)	
strongly improved	5 (1.0%)	23 (5.5%)	<0.001
improved	29 (5.9%)	82 (19.7%)	
no change	60 (12.2%)	227 (54.6%)	
worsened	315 (64.3%)	80 (19.2%)	
strongly worsened	81 (16.5%)	4 (1.0%)	
missing	1	1	

Details on the comparison of how the pandemic affected students’ mental health- related feelings are described as follows (students could select multiple answers): More students (n = 320; 63.3%) with moderate to high negative stress disclosed negative emotions including to feeling sad and/or overwhelmed and/or having poor sleep and/or negative stress as affecting their mental health compared to students (n = 90; 22.1%) with no or low negative mental health stress (p<0.001; chi-squared-test). Details can be found in Table 18.

**Table 18:** Comparison of distress and mental health-related feelings.

How was your mental health affected by the current pandemic?	Moderate to highest stress level	No or little stress level	P-value
	(n = 491)	(n = 417)	
feeling sad	10 (2.1%)	10 (2.5%)	<0.001
feeling overwhelmed	14 (2.9%)	11 (2.7%)	
poor sleep	8 (1.7%)	17 (4.2%)	
poor sleep and feeling sad	16 (3.3%)	4 (1.0%)	
lots of negative stress	33 (6.8%)	18 (4.4%)	
lots of negative stress and feeling sad	28 (5.8%)	6 (1.5%)	
lots of negative stress and feeling overwhelmed	25 (5.2%)	6 (1.5%)	
lots of negative stress, feeling overwhelmed and sad	39 (8.1%)	2 (0.5%)	
lots of negative stress and poor sleep	20 (4.1%)	7 (1.7%)	
lots of negative stress, poor sleep and feeling sad	30 (6.2%)	3 (0.7%)	
lots of negative stress, poor sleep, feeling overwhelmed	26 (5.4%)	3 (0.7%)	
lots of negative stress, poor sleep, feeling overwhelmed, feeling sad	71 (14.7%)	3 (0.7%)	
no change	16 (3.3%)	91 (22.3%)	
feeling balanced	7 (1.5%)	14 (3.4%)	
feeling relaxed, balanced, well rested and lots of positive energy	2 (0.4%)	30 (7.4%)	
other combination	137 (28.4%)	183 (44.9%)	
missing	9	9	

Table 19 provides a comparison of distress due to social distancing in students with no or low mental health-related distress compared to those with moderate to highest distress. Significant differences between the two groups’ mean were visible (mean = 4.3 (sd = 2.70) vs. 6.6 (sd = 2.86); p<0.001; t-test).

**Table 19:** Comparison of distress due to social distancing.

Level of negative stress due to social distancing	Moderate to highest stress level	No or little stress level	P-value
	(n = 491)	(n = 417)	
N	491	416	<0.001
missing	0	1	
mean	6.6	4.3	
SD	2.70	2.86	
median	7	4	
Q1—Q3	5–9	2–7	
min.—max.	0.0–10.0	0.0–10.0	

SD: Standard deviation

A detailed analysis shows significant differences in students’ level of distress due to spending most time at home: Students with moderate to highest stress level had a mean of 6.2 (sd = 2.99), comparted to students with no or little stress level (mean = 3.4; sd = 3.03; p-value<0.001; t-test). Details can be found in Table 20.

**Table 20:** Comparison of distress due to spending most time at home.

Level of negative stress due to spending most time at home	Moderate to highest stress level	No or little stress level	P-value
	(n = 491)	(n = 417)	
N	491	416	<0.001
missing	0	1	
mean	6.2	3.4	
SD	2.99	3.03	
median	7	3	
Q1—Q3	4–9	0–6	
min.—max.	0.0–10.0	0.0–10.0	

SD: Standard deviation

No significant difference in the age of students with moderate to high levels of negative stress compared to students with no or little negative stress levels related to mental health was observed (p = 0.113; t-test). Details can be found in Table 21.

**Table 21:** Comparison of distress and age.

Age	Moderate to highest stress level	No or little stress level	P-value
	(n = 491)	(n = 417)	
N	488	407	0.113
missing	3	10	
mean	23.2	22.8	
SD	4.04	3.81	
median	22	22	
Q1—Q3	21–24	20–24	
min.—max.	18.0–50.0	18.0–49.0	

SD: Standard deviation

#### 3.4.3 Group comparisons of female and male students

The following section describes an analysis of results stratified by gender and offers a group comparison of female and male students. Significantly more female students studied in Heidelberg and Ludwigshafen whereas more male students were enrolled in Mannheim (p = <0.001; chi-squared test). Details can be found in Table 22.

**Table 22:** Comparison of gender and city of university (of applied sciences).

City of university (of applied sciences)	Female (n = 472)	Male (n = 432)	P-value
Heidelberg	139 (29.4%)	105 (24.5%)	<0.001
Ludwigshafen	152 (32.2%)	74 (17.2%)	
Mannheim	181 (38.3%)	250 (58.3%)	
missing	0	3	

The following two tables (Tables 23 and 24) comparing gender and learning and education (online classes compared to face-to-face teaching) and gender and change in grades, showed that there was no significant difference between male and female students (p = 0.491 and p = 0.787; Wilcoxon test).

**Table 23:** Comparison of gender and online classes versus face-to-face teaching.

In general, how much do you think your learning and education is affected by online classes compared to face-to-face teaching?	Female (n = 472)	Male (n = 432)	P-value
strongly benefits	20 (4.3%)	12 (2.8%)	0.491
benefits	89 (18.9%)	69 (16.1%)	
no change	37 (7.9%)	62 (14.5%)	
decreases	220 (46.8%)	177 (41.3%)	
strongly decreases	104 (22.1%)	109 (25.4%)	
missing	2	3	

There was no significant difference related to the change in grades during the pandemic between male and female students.

**Table 24:** Comparison of gender and change in grades.

In general, how strongly did your grades change during the current pandemic?	Female (n = 472)	Male (n = 432)	P-value
strongly improved	6 (1.3%)	7 (1.7%)	0.787
improved	46 (10.3%)	57 (13.6%)	
no change	251 (56.0%)	212 (50.5%)	
worsened	117 (26.1%)	119 (28.3%)	
strongly worsened	28 (6.2%)	25 (6.0%)	
missing	24	12	

Comparing gender and change in mental health, 58.5% (n = 276) of female students reported a worsening of their mental health during the current pandemic compared to 46.8% (n = 202) male students (p = 0.006; Wilcoxon test). Details can be found in Table 25.

**Table 25:** Comparison of gender and change in mental health.

Did your mental health improve or worsen during the current pandemic?	Female (n = 472)	Male (n = 432)	P-value
strongly improved	14 (3.0%)	14 (3.2%)	0.006
improved	56 (11.9%)	54 (12.5%)	
no change	124 (26.4%)	162 (37.5%)	
worsened	231 (49.1%)	164 (38.0%)	
strongly worsened	45 (9.6%)	38 (8.8%)	
missing	2	0	

#### 3.4.4 Results based on the logistic regression analysis

Table 26 shows the results of the logistic regression analysis of the binary variables for mental health as the outcome variable and the baseline features as covariates. In case of categorical variables, a category is always taken as a reference and results are always set in relation to the reference.

**Table 26:** Results of logistic regression analysis with binary mental health variable as outcome variable.

Variables	Odds Ratio	95% CI	P-value
gender: male vs. female (ref.)	0.698	[0.515; 0.945]	0.020
age	1.032	[0.991; 1.074]	0.127
site: Ludwigshafen vs. Heidelberg (ref.)	1.324	[0.895; 1.957]	0.160
site: Mannheim vs. Heidelberg (ref.)	1.134	[0.776; 1.658]	0.515
studies: Health vs. Business (ref.)	0.71	[0.458, 1.101]	0.126
studies: IT vs. Business (ref.)	0.944	[0.622, 1.431]	0.785
studies: MINT/Technical vs. Business (ref.)	0.668	[0.432, 1.034]	0.070
studies: Other vs. Business (ref.)	1.175	[0.674, 2.048]	0.569
part-time working: yes vs. no (ref.)	1.253	[0.94, 1.671]	0.124
grade: good vs. very good (ref.)	1.092	[0.774, 1.542]	0.616
grade: average vs. very good (ref.)	1.162	[0.76, 1.777]	0.487
grade: poor vs. very good (ref.)	8.205	[0.971, 69.305]	0.053
semester	0.952	[0.886; 1.022]	0.174

CI: confidence interval; IT: Information technology; Ref: reference

Gender showed the only significant effect on mental health distress due to COVID-19. Male students tended to have a lower risk of moderate to high stress compared to female students (odds ratio = 0.698, CI 0.515 to 0.945). An odds ratio of <1 (here: 0.698) for men means that men have a lower risk of a high level of mental stress compared to women (these were used here as a reference). No significant age difference was observed between students with moderate to high levels of distress compared to students with little or no distress related to mental health (odds ratio = 1.032; CI 0.991 to 1.074).

In terms of students’ semester, no significant difference between students with moderate to high distress compared to students with little or no distress related to mental health was observed (odds ratio = 0.952; CI 0.886 to 1.022).

Furthermore, no significant site difference between Ludwigshafen versus Heidelberg (reference) and between Mannheim versus Heidelberg (reference) was observed between students with moderate to high negative stress compared to students with little or no negative stress related to mental health (odds ratio = 1.324; CI 0.895 to 1.957) and (odds ratio = 1.134; 0.776 to 1.658). Details are summarized in Table 26.

## 4. Discussion

The ongoing COVID-19 pandemic had severe health-related consequences. This is especially true for vulnerable groups, including university students, who were disproportionally affected by public health measures to contain the spread of the virus. These included but were not limited to unprecedented limitations of individual freedom due to social distancing which included the temporary closure of tertiary institutions with the consequence of remote learning and the ban of in person sociocultural locations.

To the best of our knowledge, this is the first study investigating the effects of the COVID-19 pandemic on university students’ physical and mental health and learning in Germany.

### 4.1 Impact on students’ physical and mental health

For students enrolled at one of the eight universities (of applied sciences) in the Rhine-Neckar region including Heidelberg, Mannheim and Ludwigshafen in Germany, this study identified a deterioration in students’ physical health and fitness as well as a worsening of students’ mental health with almost every second student disclosing to feel sad and/or overwhelmed and/or having poor sleep and/or experiencing lots of negative stress. Main stressors were due to social distancing, spending most time at home and mental health. More than half of the students expected negative outcomes related to the future due to the pandemic and many reported an increase in activities that help them relax or strengthen their mental health.

Our findings are supported by other studies reporting on reduced physical health and fitness in students during the current pandemic. There is evidence that sports activities have a moderate effect in decreasing anxiety and may improve mood and mental wellbeing [4]. Exercises performed at home, including aerobic exercises, body weight training, etc. can support a reduction in harmful physical and mental effects due to COVID-19 regulations [5]. As the winter term 2019/2020 was the first academic semester affected by COVID-19, students were more sedentary and reported more symptoms of anxiety and depression compared to previous academic semesters and subsequent academic breaks [7]. Moreover, physical exercise may stimulate positive changes in self-perception, which can be expressed in an increase in global self-esteem [37]. Furthermore, regular physical activity of two to three hours per week were positively associated with academic success [38]. Similar to respondents in this study who reported to feel sad, overwhelmed, having poor sleep and lots of negative stress, a study on college students in the United States of America identified that more than two thirds of students reported increased levels of stress and anxiety due to the COVID-19 pandemic. Several stressors including fear and concern for their own health and that of their relatives, difficulty in focusing, sleep disorders, reduced social interactions due to physical distancing and increased worries on academic performance were discovered [39]. To deal with stress and anxiety, participants sought support from others and helped themselves by introducing either negative or positive coping mechanisms [39]. A cross-sectional survey from China found that the current pandemic significantly affected the mental health of the Chinese public and that young individuals disposed of a greater risk of anxiety than older individuals [40]. During the COVID-19 outbreak in Wuhan, China, a further cross-sectional study of Chinese adults identified a prevalence of depression in almost 50%, of anxiety in almost 25% and a combination of depression and anxiety in nearly 20% of participants [41]. Thus, students might have experienced mental health problems or an aggravation of existing symptoms [42]. Further findings from North America showed an overall increase in study‐related distress in university students after the onset of the pandemic [43]. Thus, significant changes and challenges related to students’ physical and mental health were identified.

### 4.2 Gender-related differences in experiences with distress

Significant differences in gender and change in mental health were found in our study with female students reporting a worsening of their mental health during the current pandemic compared to male students. According to our findings from the logistic regression analysis, gender showed the only significant effect on mental health distress due to COVID-19 with male students having a lower risk of moderate to high stress compared to female students.

Related to students’ mental health, our results are supported by latest research from China, highlighting that women who had student status perceived a significantly higher psychological impact of COVID-19 and raised levels of stress, anxiety and depression [44]. Also, a multivariate modelling (mixed-effects logistic regression) identified that being female, having fair or poor general health status, being 18 to 24 years old, spending eight or more hours online daily and knowing someone who tested positive for COVID-19 predicted higher levels of psychological impact when risk factors were considered simultaneously [45]. A study on the impact of COVID-19 pandemic on self-reported health from the German National Cohort concluded that the pandemic and protective measures during the first wave in 2020 had implications for mental health and self-rated general health. A study on perceived stress among university students in virtual classrooms during COVID-19 in Saudi Arabia, found a moderate to high level of stress at the beginning of the pandemic with a significant correlation between high levels of stress and female sex [46]. Depression and anxiety-related symptoms increased relative to baseline in participants under 60, especially young women. The rate of moderate to severe depressive symptoms rose from 6.4% to 8.8%. The results for mental state and self-rated health deteriorated in the participants tested for SARS-CoV-2 compared to those not tested [47]. Also, a study from Croatia concluded that mental distress was stronger in women [48]. Moreover, a method to promote positive self-esteem in females was to encourage physical activity [49]. Gender-related differences could be recognized and should be taken into consideration when addressing students’ mental health.

### 4.3 Options to avoid or alleviate distress

First and foremost, sources of negative stress need to be reduced to avoid or alleviate distress. This can be achieved via the concept “locus of control”. Also, the importance of perceived togetherness and belonging should be taken into consideration. Potential relief from stressors include relaxation,mindfulness and (online) counselling services.

### 4.4 Locus of control: Importance of information and education

Locus of control is generally an important concept, but even more so during a pandemic that severely affects students’ day-to-day lives. Having control over the outcome of events can positively attribute to mental health and wellbeing. During the current pandemic, concise timely and accurate health-related information (such as the local number of infections) and appropriate precautionary measures (including hand hygiene, wearing a face mask) were associated with lower psychological effects connected to the pandemic and lower levels of stress, anxiety and depression [44]. On the other hand, chance locus of control was a prediction of moral disengagement, which was connected with less positive emotions, more negative emotions, poorer mental wellbeing and poorer general health due to less prosocial engagement [50]. Tertiary education systems can add to the education level of their students and thus provide a feeling of safety and control, especially during times of increased uncertainty.

### 4.5 The importance of togetherness and belonging

Social distancing, lockdowns and curfews might be perceived as especially challenging for students. “Social distancing” may be a misleading term referring to “physical distance”. Thus, uplifting relationships, especially by phone, video or social media solutions can be maintained during “social distancing” [51]. To counterbalance the negative effects of social distancing, everyday routines that include a healthy lifestyle, virtual social interactions and mindfulness are recommended [52]. A study on academic motivation in college freshmen revealed associations between students’ sense of class belonging and their academic self-efficacy and intrinsic motivation [21]. Thus, a student’s sense of belonging is also an important factor in academic success [21]. Social interaction showed that strengthening rich social relationships is helpful for the wellbeing of women [53]. To counteract the reduction in students’ wellbeing during periods of social isolation, those without social networks need to be supported [18]. To strengthen students’ mental health and encourage them to seek support, educational institutions might increasingly initiate contactless social gatherings. This might be a feasible and cost-efficient way to provide students with information, enhancing their perceived locus of control and feeling of togetherness.

### 4.6 Relaxation and mindfulness

We found that students increased activities to relax or strengthen their mental health. A review and meta-analysis provided evidence that “cognitive, behavioral, and mindfulness interventions” effectively reduced stress among university students [54]. Students who participated in a mindfulness-based intervention group showed lower distress even after the period of the mindfulness courses and during the examination period compared to the beginning of the study [55]. Mindfulness enhanced psychological resilience to academic distress [55]. To combine mindfulness and relaxation, yoga might be beneficial. Recent findings showed that yoga can improve somatization and mental health and thus has implications in preventing psychosomatic symptoms in healthy women [56]. There is evidence to consider yoga as a helpful, low-risk, and inexpensive addition to the treatment of stress, anxiety, depression and stress-related medical illnesses [57]. Yoga might serve as intervention in young adult health promotion [58]. Relaxation methods and the practice of mindfulness may be a low-threshold way to minimize stress, especially for the vulnerable group of university students.

### 4.7 (Online) counselling services

A systematic review and meta-analysis on the effects of mental health interventions for students in higher education showed that interventions to strengthen students’ positive mental health suggest favourable effects [59]. There are global impulses to increase the use of telehealth in response to COVID-19 [51]. Early and comprehensive support measures and behavioural plans help reduce the negative psychological effects of social isolation and might reducing the disincentive to isolate [60]. Therefore, adequate information and counselling services should be offered by education institutions to support the mental health and wellbeing of students. Sahu [61] argued that, faculties should offer current technical solutions to evade from their social isolation. Findings from China indicate that compared to those with high self-control, individuals with low self-control have increased vulnerability and more in need of psychological support to sustain mental health during the current COVID-19 outbreak [62]. New methods of consultation for mental health needs, including online services, can be supporting for patients [63]. There is a global impetus to increase the use of telehealth in response to COVID-19 [51]. In China, 24/7 web-based psychological counselling services were widely applied by mental health professionals in medical institutions, universities and academic societies. Web-based psychological self-help intervention systems have also been developed, including online cognitive-behavioural therapy for depression, anxiety and insomnia [64]. Free and inclusive (online) counselling services might help students counterbalance increased levels of distress.

### 4.8 Strengths and limitations

A strength of this study was the response rate and large amount of data gathered from the Rhine-Neckar region, including universities and universities of applied sciences with 1,246 students of which a total of 917 students completed the survey in full.

This study has methodological limitations. One was that survey questions were not based on a validated questionnaire, but a self-designed questionnaire based on a literature review. This limits regional or international comparisons. The second is that convenience sampling was applied. Convenience sampling does not produce representative results, thus the results of this study cannot be generalized to the general population. The third limitation is self-reporting as it is subject to social desirability bias. Study participants may provide more socially acceptable answers rather than being truthful. On the other hand, self-reported data are accurate when individuals understand the questions and when there is a strong sense of anonymity and little fear of negative consequences. To minimize bias caused by self-reporting, we asked questions in a short and simple style, we made study participation anonymous and we ensured that study participants did not have to fear any negative consequences from study participation.

Nevertheless, the findings contribute useful knowledge and improve understanding of key impacts and challenges for students in the region. Yet, these findings are relevant for both a regional and international audience as students experience similar physical and mental health challenges and potential negative consequences for their academic performance due to temporary lockdowns and distance learning during this pandemic.

## 5. Conclusion

Even though the COVID-19 pandemic is primarily a threat to short and long-term physical health, experiences from 917 students from eight universities in the Rhine-Neckar region in Germany showed, that besides students’ physical health, their mental health, social wellbeing and academic performance was also negatively affected by the pandemic. Social distancing, spending most time at home and e-learning via video conferences were perceived as considerable stressors for students. This study identified significant differences related to students’ mental health based on a grouped comparison of students with no or little distress compared to students with moderate to high levels of distress. Students with moderate to high distress, compared to students with no or little distress, declared more often that their physical health and fitness worsened and their mental health deteriorated. A logistic regression analysis found that gender showed a significant effect on mental health distress due to COVID-19. Male students were identified to have a lower risk of moderate to high levels of distress compared to female students.

Tailored, low-threshold initiatives for students, ideally taking the different needs of male and female students into consideration, that contain multidimensional approaches focusing on students’ physical and mental health as well as their academic needs may alleviate students’ distress caused by the current pandemic.

The findings allow to learn how to address university students’ perceived needs in order to offer low-threshold responses at an early stage during unprecedented and highly challenging situations and to offer an information base to identify which further data is required to address students’ health and academic performance. Also, implications for further studies are needed to shed light on the long-term effects of COVID-19 on students’ physical and mental health and effects on learning and grades during the COVID-19 pandemic.

## Supporting information

S1 Table (DOCX)

## Abbreviations

CI: Confidence interval
COVID-19: Coronavirus disease 2019
IQR: Interquartile range
IT: Information technology
MINT: Mathematics, Informatics, Natural Sciences and Technology
OR: Odds ratio
SARS-CoV-2: Severe acute respiratory syndrome coronavirus 2
SD: Standard deviation
