# Supplementary material for: “Effects of the COVID-19 pandemic on university students’ physical health, mental health and learning, a cross-sectional study including 917 students from eight universities in Germany”
Source: PLoS One. 2022 Aug 31;17(8):e0273928. doi: 10.1371/journal.pone.0273928 (PMC9432688; doi:10.1371/journal.pone.0273928)
Supplement: S1 Table — (DOCX) [file pone.0273928.s001.docx]

| **Descriptive analysis** | |
| --- | --- |
|  | **Total** |
|  | **(n = 917)** |
|  |  |
| Did your physical health improve or worsen during the pandemic? (Phys_h) |  |
| - Strongly improved | 31 (3.4%) |
| - improved | 134 (14.6%) |
| - no change | 398 (43.5%) |
| - worsened | 307 (33.6%) |
| - strongly worsened | 45 (4.9%) |
| - Missing | 2 |
|  |  |
|  |  |
| Did your overall fitness increase or decrease during the current pandemic? (Fitness) |  |
| - Strongly increased | 56 (6.1%) |
| - increased | 179 (19.6%) |
| - no change | 219 (23.9%) |
| - decreased | 342 (37.4%) |
| - strongly decreased | 119 (13.0%) |
| - Missing | 2 |
|  |  |
| Did your eating habits change during the pandemic? (Eat_combi_short) |  |
| - No, I did not change my eating habits | 198 (21.6%) |
| - Yes, I cooked more | 73 (8.0%) |
| - Yes, I ate more sweets | 38 (4.1%) |
| - Yes, I ate more sweets and cooked more | 19 (2.1%) |
| - Yes, I ate more healthy and fresh food | 63 (6.9%) |
| - Yes, I ate more healthy and fresh foodand ooked more | 50 (5.5%) |
| - Yes, I ate less | 55 (6.0%) |
| - Yes, I ate more | 94 (10.3%) |
| - Yes, I ate and cooked more | 38 (4.1%) |
| - Yes, I ate more (also sweets) | 53 (5.8%) |
| - Yes, I ate more (also sweets) and cooked more | 51 (5.6%) |
| - other combination | 184 (20.1%) |
| - Missing | 1 |
|  |  |
| How was your mental health affected by the current pandemic? (Text answers included) (Mental_combi_short) |  |
| - feeling sad | 20 (2.2%) |
| - feeling overwhelmed | 25 (2.8%) |
| - poor sleep | 25 (2.8%) |
| - poor sleep and feeling sad | 20 (2.2%) |
| - lots of negative stress | 52 (5.8%) |
| - lots of negative stress and feeling sad | 34 (3.8%) |
| - lots of negative stress and feeling overwhelmed | 31 (3.5%) |
| - lots of negative stress, feeling overwhelmed and sad | 41 (4.6%) |
| - lots of negative stress and poor sleep | 28 (3.1%) |
| - lots of negative stress, poor sleep and feeling sad | 33 (3.7%) |
| - lots of negative stress, poor sleep, feeling overwhelmed | 29 (3.2%) |
| - lots of negative stress, poor sleep, feeling overwhelmed, feeling sad | 74 (8.3%) |
| - no change | 109 (12.2%) |
| - feeling balanced | 21 (2.3%) |
| - Feeling relaxed, balanced, well rested and lots of positive energy | 32 (3.6%) |
| - other combination | 322 (35.9%) |
| - Missing | 21 |
|  |  |
| How was your mental health affected by the current pandemic? (Mental_combi_notext_short) |  |
| - feeling sad | 22 (2.5%) |
| - feeling overwhelmed | 29 (3.2%) |
| - poor sleep | 26 (2.9%) |
| - poor sleep and feeling sad | 20 (2.2%) |
| - lots of negative stress | 56 (6.3%) |
| - lots of negative stress and feeling sa | 38 (4.3%) |
| - lots of negative stress and feeling overwhelmed | 33 (3.7%) |
| - lots of negative stress, feeling overwhelmed and sad | 45 (5.0%) |
| - lots of negative stress and poor sleep | 30 (3.4%) |
| - lots of negative stress, poor sleep and feeling sad | 37 (4.1%) |
| - lots of negative stress, poor sleep, feeling overwhelmed | 30 (3.4%) |
| - lots of negative stress, poor sleep, feeling overwhelmed, feeling sad | 79 (8.8%) |
| - no change | 120 (13.4%) |
| - feeling balanced | 22 (2.5%) |
| - Feeling relaxed, balanced, well rested and lots of positive energy | 36 (4.0%) |
| - other combination | 270 (30.2%) |
| - Missing | 24 |
|  |  |
| Did your mental health improve or worsen during the current pandemic? (Ment_health) |  |
| - Strongly improved | 28 (3.1%) |
| - improved | 111 (12.1%) |
| - no change | 290 (31.7%) |
| - worsened | 400 (43.8%) |
| - strongly worsened | 85 (9.3%) |
| - Missing | 3 |
|  |  |
|  |  |
| Level of negative stress due to physical health (Stress_ph) |  |
| - N | 911 |
| - Missing | 6 |
| - Mean | 3.5 |
| - SD | 2.88 |
| - Median | 3 |
| - Q1 -- Q3 | 1 -- 6 |
| - Min. -- Max. | 0.0 -- 10.0 |
|  |  |
| Level of negative stress due to mental health (Stress_mh) |  |
| - N | 908 |
| - Missing | 9 |
| - Mean | 4.6 |
| - SD | 3.09 |
| - Median | 5 |
| - Q1 -- Q3 | 2 -- 7 |
| - Min. -- Max. | 0.0 -- 10.0 |
|  |  |
| Level of negative stress due to social distancing (Stress_dist) |  |
| - N | 910 |
| - Missing | 7 |
| - Mean | 5.6 |
| - SD | 3.00 |
| - Median | 6 |
| - Q1 -- Q3 | 3 -- 8 |
| - Min. -- Max. | 0.0 -- 10.0 |
|  |  |
| Level of negative stress due to closing student dorms (Stress_dorm) |  |
| - N | 902 |
| - Missing | 15 |
| - Mean | 1.7 |
| - SD | 2.89 |
| - Median | 0 |
| - Q1 -- Q3 | 0 -- 3 |
| - Min. -- Max. | 0.0 -- 10.0 |
|  |  |
| Level of negative stress due to closing shops that are not vital (Stress_shop) |  |
| - N | 909 |
| - Missing | 8 |
| - Mean | 2.2 |
| - SD | 2.56 |
| - Median | 1 |
| - Q1 -- Q3 | 0 -- 4 |
| - Min. -- Max. | 0.0 -- 10.0 |
|  |  |
|  |  |
| Level of negative stress due to closing sports facilities (Stress_sport) |  |
| - N | 909 |
| - Missing | 8 |
| - Mean | 4.4 |
| - SD | 3.47 |
| - Median | 5 |
| - Q1 -- Q3 | 0 -- 7 |
| - Min. -- Max. | 0.0 -- 10.0 |
|  |  |
| Level of negative stress due to spending most time at home (Stress_home) |  |
| - N | 911 |
| - Missing | 6 |
| - Mean | 5.0 |
| - SD | 3.31 |
| - Median | 5 |
| - Q1 -- Q3 | 2 -- 8 |
| - Min. -- Max. | 0.0 -- 10.0 |
|  |  |
| Level of negative stress due to not meeting family (Stress_fam) |  |
| - N | 905 |
| - Missing | 12 |
| - Mean | 4.2 |
| - SD | 3.58 |
| - Median | 4 |
| - Q1 -- Q3 | 0 -- 7 |
| - Min. -- Max. | 0.0 -- 10.0 |
|  |  |
|  |  |
| Level of negative stress due to physical health [cat] (Stress_ph_cat) |  |
| - 0 = no stress | 227 (24.9%) |
| - 1 | 66 (7.2%) |
| - 2 | 105 (11.5%) |
| - 3 | 87 (9.5%) |
| - 4 | 69 (7.6%) |
| - 5 = medium stress | 117 (12.8%) |
| - 6 | 82 (9.0%) |
| - 7 | 69 (7.6%) |
| - 8 | 47 (5.2%) |
| - 9 | 20 (2.2%) |
| - 10 = highest level of stress | 22 (2.4%) |
| - Missing | 6 |
|  |  |
| Level of negative stress due to mental health [cat] (Stress_mh_cat) |  |
| - 0 = no stress | 140 (15.4%) |
| - 1 | 56 (6.2%) |
| - 2 | 77 (8.5%) |
| - 3 | 77 (8.5%) |
| - 4 | 67 (7.4%) |
| - 5 = medium stress | 100 (11.0%) |
| - 6 | 79 (8.7%) |
| - 7 | 123 (13.5%) |
| - 8 | 98 (10.8%) |
| - 9 | 45 (5.0%) |
| - 10 = highest level of stress | 46 (5.1%) |
| - Missing | 9 |
|  |  |
| Level of negative stress due to social distancing [cat] (Stress_dist_cat) |  |
| - 0 = no stress | 70 (7.7%) |
| - 1 | 38 (4.2%) |
| - 2 | 69 (7.6%) |
| - 3 | 86 (9.5%) |
| - 4 | 48 (5.3%) |
| - 5 = medium stress | 117 (12.9%) |
| - 6 | 79 (8.7%) |
| - 7 | 107 (11.8%) |
| - 8 | 133 (14.6%) |
| - 9 | 74 (8.1%) |
| - 10 = highest level of stress | 89 (9.8%) |
| - Missing | 7 |
|  |  |
| Level of negative stress due to closing student dorms [cat] (Stress_dorm_cat) |  |
| - 0 = no stress | 614 (68.1%) |
| - 1 | 23 (2.5%) |
| - 2 | 35 (3.9%) |
| - 3 | 27 (3.0%) |
| - 4 | 21 (2.3%) |
| - 5 = medium stress | 61 (6.8%) |
| - 6 | 23 (2.5%) |
| - 7 | 26 (2.9%) |
| - 8 | 33 (3.7%) |
| - 9 | 15 (1.7%) |
| - 10 = highest level of stress | 24 (2.7%) |
| - Missing | 15 |
|  |  |
| Level of negative stress due to closing shops that are not vital [cat] (Stress_shop_cat) |  |
| - 0 = no stress | 391 (43.0%) |
| - 1 | 84 (9.2%) |
| - 2 | 95 (10.5%) |
| - 3 | 88 (9.7%) |
| - 4 | 53 (5.8%) |
| - 5 = medium stress | 94 (10.3%) |
| - 6 | 38 (4.2%) |
| - 7 | 29 (3.2%) |
| - 8 | 16 (1.8%) |
| - 9 | 5 (0.6%) |
| - 10 = highest level of stress | 16 (1.8%) |
| - Missing | 8 |
|  |  |
| Level of negative stress due to closing sports facilities [cat] (Stress_sport_cat) |  |
| - 0 = no stress | 230 (25.3%) |
| - 1 | 45 (5.0%) |
| - 2 | 61 (6.7%) |
| - 3 | 61 (6.7%) |
| - 4 | 46 (5.1%) |
| - 5 = medium stress | 105 (11.6%) |
| - 6 | 55 (6.1%) |
| - 7 | 86 (9.5%) |
| - 8 | 89 (9.8%) |
| - 9 | 55 (6.1%) |
| - 10 = highest level of stress | 76 (8.4%) |
| - Missing | 8 |
|  |  |
| Level of negative stress due to spending most time at home [cat] (Stress_home_cat) |  |
| - 0 = no stress | 143 (15.7%) |
| - 1 | 45 (4.9%) |
| - 2 | 70 (7.7%) |
| - 3 | 76 (8.3%) |
| - 4 | 56 (6.1%) |
| - 5 = medium stress | 99 (10.9%) |
| - 6 | 74 (8.1%) |
| - 7 | 85 (9.3%) |
| - 8 | 105 (11.5%) |
| - 9 | 71 (7.8%) |
| - 10 = highest level of stress | 87 (9.5%) |
| - Missing | 6 |
|  |  |
| Level of negative stress due to not meeting family [cat] (Stress_fam_cat) |  |
| - 0 = no stress | 251 (27.7%) |
| - 1 | 53 (5.9%) |
| - 2 | 74 (8.2%) |
| - 3 | 50 (5.5%) |
| - 4 | 50 (5.5%) |
| - 5 = medium stress | 87 (9.6%) |
| - 6 | 59 (6.5%) |
| - 7 | 59 (6.5%) |
| - 8 | 72 (8.0%) |
| - 9 | 57 (6.3%) |
| - 10 = highest level of stress | 93 (10.3%) |
| - Missing | 12 |
|  |  |
|  |  |
| Level of negative stress due to physical health [binary] (Stress_ph_bi) |  |
| - moderate to highest stress level | 357 (39.2%) |
| - no or little stress level | 554 (60.8%) |
| - Missing | 6 |
|  |  |
| Level of negative stress due to mental health [binary] (Stress_mh_bi) |  |
| - moderate to highest stress level | 491 (54.1%) |
| - no or little stress level | 417 (45.9%) |
| - Missing | 9 |
|  |  |
| Level of negative stress due to social distancing [binary] (Stress_dist_bi) |  |
| - moderate to highest stress level | 599 (65.8%) |
| - no or little stress level | 311 (34.2%) |
| - Missing | 7 |
|  |  |
| Level of negative stress due to closing student dorms [binary] (Stress_dorm_bi) |  |
| - moderate to highest stress level | 182 (20.2%) |
| - no or little stress level | 720 (79.8%) |
| - Missing | 15 |
|  |  |
| Level of negative stress due to closing shops that are not vital [binary] (Stress_shop_bi) |  |
| - moderate to highest stress level | 198 (21.8%) |
| - no or little stress level | 711 (78.2%) |
| - Missing | 8 |
|  |  |
|  |  |
| Level of negative stress due to closing sports facilities [binary] (Stress_sport_bi) |  |
| - moderate to highest stress level | 466 (51.3%) |
| - no or little stress level | 443 (48.7%) |
| - Missing | 8 |
|  |  |
| Level of negative stress due to spending most time at home [binary] (Stress_home_bi) |  |
| - moderate to highest stress level | 521 (57.2%) |
| - no or little stress level | 390 (42.8%) |
| - Missing | 6 |
|  |  |
| Level of negative stress due to not meeting family [binary] (Stress_fam_bi) |  |
| - moderate to highest stress level | 427 (47.2%) |
| - no or little stress level | 478 (52.8%) |
| - Missing | 12 |
|  |  |
|  |  |
| Did you increase activities that help you relax or strengthen your mental health? (Relax) |  |
| - Very strongly | 47 (5.1%) |
| - strongly | 114 (12.5%) |
| - a little | 356 (39.0%) |
| - very little | 109 (11.9%) |
| - not at all | 287 (31.4%) |
| - Missing | 4 |
|  |  |
|  |  |
